# How to Solve the Problems of Docking into a Symmetric Binding Site: The Example of the hERG Channel

**DOI:** 10.3797/scipharm.1307-01

**Published:** 2013-07-31

**Authors:** Andrea Schiesaro, Lars Richter, Gerhard F. Ecker

**Affiliations:** University of Vienna, Department of Medicinal Chemistry, Althanstraße 14, 1090, Vienna, Austria.

**Keywords:** Symmetric Binding site, Docking, Duplicate poses, hERG

## Abstract

Many proteins, such as the hERG K^+^ channel or the HIV-1 protease, have a high degree of rotational symmetry. If the binding site of a ligand is composed of symmetrical subunits, the analysis of the docking poses of ligands is quite challenging. In the case of hERG, the four-fold symmetry of the entire channel is fully reflected in the binding site, which allows up to four poses with different coordinates of the ligand, but an identical interaction pattern. In light of our docking studies into the hERG potassium channel, we developed an algorithm (ROTALI) to detect the poses that are duplicates due to the symmetry of the channel. This led to a reduction in the number of poses to be considered in the subsequent steps by up to 52%.

## Introduction

The hERG potassium channel plays an important role in the third phase of heart repolarization, where the most important current is the rapid inward rectifier current (Ikr). Ikr is created by a flow of potassium ions through the cell membrane, caused by the opening of the hERG channel [1]. Interactions with this phase of repolarization might prolong the QT time and evolve into the long QT syndrome (LQTS). The LQTS is correlated with a cardiac arrhythmia called Torsade de Pointes (TdP), which could potentially be lethal. Biological studies demonstrated the correlation between the LQTS and hERG blockage, which renders the hERG channel an antitarget. In the past few decades, many drugs were withdrawn from the market due to unwanted interactions with the hERG channel [2–4]. The strong relationship between the LQTS and inhibition of the hERG channel resulted in great interest in the molecular basis of the drug-channel interaction, which led to the development of various *in silico* models capable of predicting potential hERG blockers.

The hERG channel has a homotetrameric architecture. Each of the four monomers contains six transmembrane domains (S1–S6). In protein homology models, generally only the S5-P-S6 segment of one subunit is modeled, while the other three subunits are just copies thereof [1]. Hence, only the parts that define the binding site, the selectivity filter, the central cavity, and the inner pore, are included in the model. This operation leads to a four-fold symmetry, which has consequences when docking small molecules into this model. Thus, when a docking operation is performed in a symmetric binding site, it is possible to obtain the same pose in four different directions. Usual docking algorithms cannot detect these poses as duplicates due to the placement in different directions; they have high RMSD values, even if the interaction pattern is the same. Thus, in the framework of our structure-based design studies to probe the trapped/non-trapped behaviour of propafenone analogs [5], we developed a procedure (ROTALI – ROTation and ALIgnment), which first aligns the poses, and then deletes the duplicates caused by the symmetric binding site.

## Results and Discussion

The symmetry problem can be tackled in two ways. The first possible solution comprises a rigid body rotation of the complexes around the internal symmetry axis [6]. It is possible to apply this strategy also in non-symmetric hERG channels, for example after molecular dynamics simulations. In our approach, only the ligand is rotated and the coordinates of the channel are kept fixed. This method can be used only if the protein, in this case the hERG channel, is symmetric. This approach is computationally less expensive than the first method mentioned, because only the ligand is rotated and not the entire complex. Furthermore, with this operation the visual inspection of the pose and the identification of the possible common binding modes are facilitated, as the protein stays fixed.

**Fig. 1:**
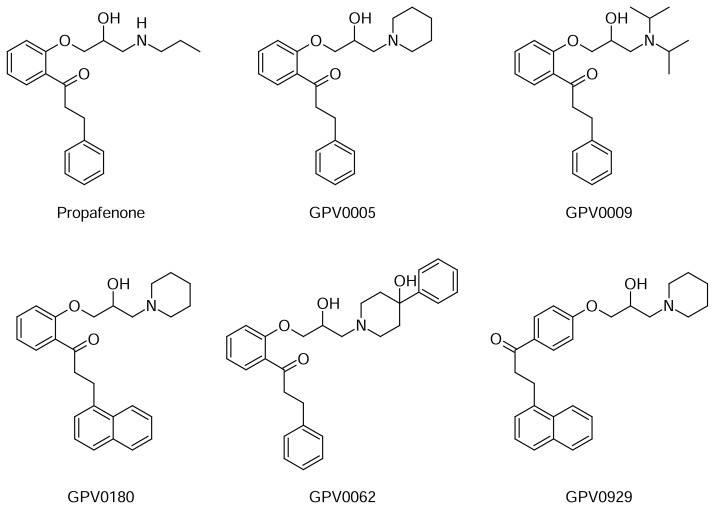
Propafenone derivatives docked into the hERG channel

**Fig. 2:**
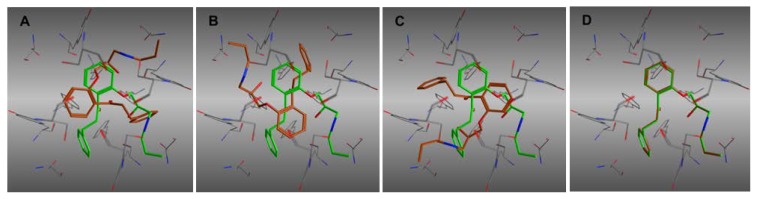
(A) The pose (brown colored) is loaded and compared to the reference pose (green colored) and the RMSD0 is calculated (B), (C), and (D). The pose is rotated 90° every time and the RMSD90, RMSD180, and RMSD270 are then calculated.

Several studies demonstrated that propafenone and its derivatives are hERG blockers, and that some of them are trapped in the hERG channel in the closed state [5, 7–10]. Thus, as a use case we docked a set of propafenone analogs (Fig. 1) into a homology model of the hERG channel in the closed state. We selected 100 poses per ligand according to the scoring function, which in total led to 600 poses. Subsequently, the svl script ROTALI was used to align the first 100 poses per ligand ranked according to the scoring function, and to calculate the RMSD matrix for detection of the duplicates. First, the symmetry axis of the protein was defined and used as a rotational axis for the docked poses. The axis was defined by the centroid of the four Cα atoms of G669 (top of channel) and the centroid of the four Cα atoms of N598 (bottom of channel). Based on this axis, the rigid body rotations of the poses were performed by implementing the MOE built-in function “rot3d_Rotation” in a svl script. The rotational angle was set to 90° in order to reflect the four-fold symmetry of the hERG binding site, leading to four poses per original pose (0°, 90°, 180°, and 270°). So each pose was quadruplicated, laying the basis for calculating the RMSD values of the poses that fall into quadrants different from the original pose. The reference pose to which all the others are compared and aligned is the first one of the database. The original placement of the second pose is compared to the reference one and the RMSD0° is calculated. Subsequently, the second pose is rotated three times and for every rotation, the RMSD value is calculated (RMSD90°, RMSD180°, and RMSD270°) (Fig. 2). The lowest value of RMSD is written in an RMSD matrix, because it corresponds to the value of the “rotational pose” that is aligned or at least “adjusted” to the reference pose. When all the poses are compared to the first one, the reference pose changes to the second one and all the operations are repeated again. The program ends when each pose has been taken as a reference. Subsequently, all poses with an RMSD value lower than 0.1 Å are considered as duplicates, which leads to a remarkable reduction of poses (Tab 1).

The percentage of duplicates deleted using ROTALI varies from a minimum of 18% for GPV0009 to a maximum of 52% for GPV0005. The differences in the percentage of duplicates between individual propafenones might be due to the bulkiness and the rigidity of the substituent attached to the protonated nitrogen. In GPV0005, the substituent is small and the fact that it is a ring limits the number of different conformations that it can adopt in the binding site, explaining the high percentage of duplicates due to symmetry. In contrast, the substituent at the nitrogen atom in GPV0009 is bulkier and more flexible than the one in GPV0005, allowing a higher number of different conformations in the binding site, especially when taking the rotamers of the isopropyl groups into consideration.

These results highlight the difficulties that arise when analyzing the poses obtained by docking into a symmetric binding site. This is not only a visual problem, but it heavily affects subsequent RMSD-based clustering of poses according to their common scaffold. The importance of taking the symmetry of the hERG potassium channel into account has also been pointed out in a recent study [11]. With ROTALI, we present an easy to execute script which allows the identification of duplicates and the alignment of poses in symmetric binding sites.

## Experimental

### Docking Protocols

The closed state of the hERG channel homology model as previously described [5], was prepared with the program Protonate 3D of MOE2012.10 [12].

The MOE suite was used to build propafenone, GPV0005, GPV0009, GPV0062, GPV0180, and GPV0929 in the R configuration with the nitrogen atom protonated [8]. Subsequently, the molecular structures were energy-minimized using the MMFF94x force field. A systematic search analysis was then performed, retaining the 100 most diverse conformations considering the potential energy and the RMSD value, per ligand and docked into the homology model of the hERG channel.

The binding site was defined by the atoms of the amino acids Ser624, Ser649, Tyr652, and Phe656 of each subunit. The Alpha PMI placement method and the minimization of the poses according to the MMFF94x force field were used to obtain better performance of the docking program (personal communication from Dr. Wolfram Altenhofen, Chemical Computing Group). For every run, 20 poses per conformation were retained and minimized by the force field, generating more than 1297 conformations per ligand. All the other parameters were left in their default values. The top-ranked 100 poses per ligand were considered for further evaluation.

## Figures and Tables

**Tab. 1:** Performance of ROTALI to detect duplicates. The performance is calculated considering the first 100 poses ranked according to the London dG scoring function

Ligands	Number of starting poses	Number of duplicates	% of duplicates
Propafenone	100	43	43%
GPV0005	100	52	52%
GPV0009	100	18	18%
GPV0062	100	43	43%
GPV0180	100	42	42%
GPV0929	100	36	36%
